# Advocacy for Better Integration and Use of Child Health Indicators for Global Monitoring

**DOI:** 10.9745/GHSP-D-23-00181

**Published:** 2023-12-22

**Authors:** Kathleen Strong, Jennifer Harris Requejo, Sk Masum Billah, Joanna Schellenberg, Melinda Munos, Marzia Lazzerini, Ambrose Agweyu, Cynthia Boschi-Pinto, Sayaka Horiuchi, Abdoulaye Maiga, Ralf Weigel, Zeina Jamaluddine, Maureen Black, Frances Aboud, Emma Sacks

**Affiliations:** a Department of Maternal, Newborn, Child and Adolescent Health and Aging, World Health Organization, Geneva, Switzerland.; b The World Bank Group, Global Financing Facility, Washington, DC, USA.; c Maternal and Child Health Division, icddr,b, Dhaka, Bangladesh.; d London School of Hygiene and Tropical Medicine, London, United Kingdom.; e Johns Hopkins Bloomberg School of Public Health, Baltimore, MD, USA.; f Institute for Maternal and Child Health IRCCS Burlo Garofolo, Trieste, Italy.; g Health Services Unit, KEMRI-Wellcome Trust Research Programme, Nairobi, Kenya.; h University Federal Fluminense, Rio de Janeiro, Brazil.; i Center for Birth Cohort Studies, University of Yamanashi, Yamanashi, Japan.; j Witten/Herdecke University, Witten, Germany.; k American University of Beirut, Beirut, Lebanon.; l Department of Pediatrics and Department of Epidemiology and Public Health, University of Maryland School of Medicine, Baltimore, MD, USA; RTI International, Research Triangle Park, NC, USA.; m McGill University, Montreal, Canada.; n Consultant, Child Health Accountability Tracking Technical Advisory Group, Baltimore, MD, USA.

## Abstract

Making better use of harmonized indicators to monitor child health and well-being at the global level will avoid duplicative monitoring and evaluation exercises, improve evidence-based programming, and preserve resources that can be used to improve the quality of national data collection platforms.

## Key Messages

- Standard validated indicators that have been recommended by normative agencies exist and should be used as appropriate for monitoring child health outcomes.
- Vertical child health monitoring and evaluation approaches can distort the prioritization of health issues at the country level and may skew national resource allocation.
- A focus on building country capacity to improve child health data collection and analysis can minimize the need for complex statistical methods to estimate national values.

## INTRODUCTION

Child survival has improved dramatically over the past 2 decades through a variety of targeted interventions.^1^^,^^2^ Yet, for 54 countries, achieving the Sustainable Development Goal (SDG) for mortality among children aged younger than 5 years of at least as low as 25 deaths per 1,000 live births by 2030 remains out of reach. Meanwhile, for other countries that have achieved the goal, the child health agenda has shifted from survival to areas such as early childhood development, injuries, and noncommunicable diseases.^3^ The divide between countries that are meeting survival targets and those left behind has prompted a reevaluation of approaches to improving child health and well-being, including monitoring and measurement efforts.^3^^–^^5^ The diverse programming needs of countries have produced a steady increase in the number of organizations collecting and reporting on data to monitor child health and well-being.^3^ Although these efforts have helped raise the visibility of child health, they have inadvertently contributed to nonstandard data collection approaches and widespread use of inconsistently defined indicators, which has made tracking the global progress of child health over time and within and across countries challenging.^6^

Advocacy for better understanding and use of data, as well as improving data availability and quality, has been an ongoing refrain in the maternal, newborn, child, and adolescent health space over the years.^3^^–^^9^ In support of improving data availability, core indicator sets have been defined for many health and well-being outcomes and impacts.^4^^,^^10^ These indicator sets balance the conflicting needs for comprehensive but succinct information on child health outcomes that are relevant to all countries and feasible to collect in a timely manner and at a reasonable cost.^5^ We take this opportunity to reinforce the call for a systematic response to measuring child health outcomes at the global level in which national governments, international organizations, and donors work together to standardize reporting on child health and well-being outcomes through global accountability mechanisms like the SDGs. Making better use of harmonized indicators to monitor child health and well-being at the global level will avoid duplicative monitoring and evaluation (M&E) exercises, improve evidence-based programming, and preserve resources that can be used to improve the quality of national data collection platforms. We describe the strengths and limitations of different data types for indicators to track progress toward global targets for child health and well-being and make recommendations for how to best use existing harmonized indicators for global monitoring.

Making better use of harmonized indicators to monitor child health and well-being at the global level will avoid duplicative M&E exercises, improve evidence-based programming, and preserve resources that can be used to improve the quality of national data collection platforms.

## GLOBAL ACCOUNTABILITY MECHANISMS FOR IMPROVING CHILD HEALTH AND WELL-BEING

The SDGs and the Global Strategy for Women's, Children's and Adolescents' Health (2016–2030) have broad agendas for improving health and well-being.^4^^,^^11^ These include using a life course approach to monitor changes in health outcomes and reemphasizing primary health care as well as universal health coverage.^11^ The SDGs and their targets were set with the idea of empowering countries to report on their progress using strengthened national information systems. However, in an era where global donors demand immediate measurement results, regardless of data quality challenges, many countries struggle with collecting and analyzing data across multiple program areas. Multiple donor initiatives, often focused on specific diseases, have left some countries with fragmented health information systems and workforces overburdened due to reporting pressures. We suggest that a set of well-defined priority indicators that are feasible to measure should be used for global monitoring and for comparing progress across countries and time.

To aid global efforts to measure progress in child survival, development, and well-being consistently, the World Health Organization and UNICEF jointly convened the Child Health Accountability Tracking (CHAT) Technical Advisory Group in 2018. Children aged 1 month to 9 years have been the focus of CHAT's measurement efforts. Over its first years, CHAT mapped the child health and well-being indicators included in global accountability initiatives, prioritized a core set of standard indicators on child health and well-being, and proposed a research agenda to address identified gaps.^12^ The 26 recommended indicators (Tables 1 and 2) relate to the leading causes of child death, disease, disability, or injury within the age range of 1 month to 9 years and have global consensus around definitions and data collection methods.^12^ These indicators are not new; they have been discussed and promoted in numerous fora and are notable for being feasible (i.e., most countries have a way to capture the data) and their definitions stable so that trends can be tracked over time. We argue that these indicators can be used universally by all global and some national measurement efforts with the aim of making child health impact and outcome monitoring more rigorous and systematic over time. A standard set of harmonized indicators to monitor child health outcomes becomes a powerful tool for assessing global and country progress and for capturing when a country achieves survival goals, signaling the need to focus on other aspects of child health and well-being.

**TABLE 1.** CHAT Core Indicators With Source, Data Type, and Status as Sustainable Development Goals or Every Women Every Child Global Strategy Indicators

CHAT Core Indicator Name^a^	CHAT Recommended Definition	Country Data Source	Country Comparable Estimates?	SDG Indicator?	EWEC GS Indicator?
Under-5 mortality rate	Probability of dying between birth and exactly 5 years of age, per 1,000 livebirths	CRVS, population-based surveys	UN-IGME (annual)	3.2.1	Yes
Older child mortality rate (5–9 years)	Probability of dying at age 5 to 9 years expressed per 1,000 children aged 5	CRVS, population-based surveys	UN-IGME (annual)	No	No
Causes of death in children under 5 and 5 to 9 years	Age specific death rates by cause as defined by ICD-11	CRVS, population-based surveys	Maternal and Child Epidemiology Estimation Group, WHO Global Health Estimates	No	No
Wasting prevalence in children under 5	% wasted (moderate and severe) children aged 0–59 months (moderate=weight for height below -2 standard deviation from the median of the WHO Child Growth Standards; severe=weight for height below -3 standard deviations from the median of the WHO Child Growth Standards)	Population-based surveys; facility data	UNICEF/WHO/World Bank Joint Child Malnutrition Estimates	2.2.2	Yes
Overweight prevalence in children under 5^b^	% overweight children aged under 5 years (overweight= weight for height >+2 standard deviation from the median of the WHO Child Growth Standards)	Population-based surveys, facility data	UNICEF/WHO/World Bank Joint Child Malnutrition Estimates	2.2.2	Yes
Stunting prevalence among children under 5	% stunted (moderate and severe) children aged 0–59 months (moderate=height-for-age below -2 standard deviations from the WHO Child Growth Standards median; severe=height-for-age below -3 standard deviations from the WHO Child Growth Standards median)	Population-based surveys, facility data	UNICEF/WHO/World Bank Joint Child Malnutrition Estimates	2.2.1	Yes
Percentage of children under 5 years of age who are developmentally on track in health, learning, and psychosocial well-being, by sex (ECDI2030)	Proportion of children under 5 years of age who are developmentally on track in health, learning, and psychosocial well-being is currently being measured by the percentage of children aged 24–59 months who are developmentally on-track in at least 3 of the following 4 domains: literacy-numeracy, physical, socio-emotional, and learning.	UNICEF Multiple Indicator Cluster Surveys	No	4.2.1	Yes
Exclusive breastfeeding	Proportion of children aged 0–5 months who are exclusively fed with breast milk	National and other surveys	No	No	Yes
Vitamin A supplementation (full coverage)	% children aged 6–59 months who received 2 age-appropriate doses of vitamin A in the past 12 months	National and other surveys, facility data	UNICEF global nutrition database based on administrative reports from countries	No	No
Full vaccination coverage (immunization according to national schedule)	Proportion of the target population covered by all vaccines included in their national program	National and other surveys, facility data	WHO and UNICEF Estimates of National Immunization Coverage (annual)	3.b.1	Yes
Measles vaccination	% children who have received 2 doses of measles containing vaccine in a given year, according to the nationally recommended schedule	National and other surveys, facility data	WHO and UNICEF Estimates of National Immunization Coverage (annual)	No	Yes
Care-seeking for children with symptoms of acute respiratory infection	% children aged under 5 years with acute respiratory infection (cough and difficult breathing not due to a problem from a blocked nose) in the previous 2 weeks taken to an appropriate health facility or provider	National and other surveys, facility data	No	As part of 3.8.1	Yes
Care-seeking for fever in children under the age of 5	% children aged under 5 years with fever in the previous 2 weeks taken to an appropriate health facility or provider	National and other surveys, facility data	No	No	No
Diarrhea treatment (ORS and zinc)	% children aged under 5 years with diarrhea in the last 2 weeks receiving ORS (fluids made from ORS packets or prepackaged ORS fluids) and zinc supplement	National and other surveys, facility data	No	No	Yes
Maltreatment, harsh punishment by caregivers	Proportion of children aged 1–17 years who experienced any physical punishment and/or psychological aggression by caregivers in the past month	UNICEF Multiple Indicator Cluster Surveys capture this indicator for children aged 1 to 14 years	No	16.2.1	No
Neural tube defect (prevalence)	Prevalence of disorders that occur during gestation, involving specific elements of the neural tube; consensus needed on a definition of prevalence for children younger than 5 years and for children aged 5–9 years	National birth defect registries, facility data	WHO and partners burden of birth defects estimates (expected 2024)	No	No
Uncorrected refractive error (prevalence)	Prevalence of refractive errors (eye disorders impeding the full development of good visual function) that have not been corrected; consensus needed on a definition of prevalence for children younger than 5 years and for children aged 5–9 years	Special surveys	No	No	No
Asthma (prevalence)	% of children younger than 5 years and children aged 5–9 years with asthma	Special surveys, facility data	Estimates from WHO, IHME, and others	No	No
Anemia prevalence in children	% of children aged 6−59 months with a hemoglobin concentration of <110 g/L, adjusted for altitude	Special surveys, facility data	WHO Global Database on Anemia	No	No
Road traffic accidents	Years of life lost to disability due to road traffic accidents among children aged 0–9 years	Special surveys, facility data, and road traffic authority/police reports	WHO Global Health Estimates, Child and Adolescent Cause of Death Estimates (CA-CODE; WHO and partners)	No	No

Abbreviations: CHAT, Child Health Accountability Tracking; CRVS, civil registration and vital statistics; ECDI2030, Early Childhood Development Index 2030; EWEC GS, Every Woman Every Child Global Strategy; ICD-11, International Classification of Diseases 11th Revision; IHME, Institute for Health Metrics and Evaluation; ORS, oral rehydration solution; SDG, Sustainable Development Goal; UN-IGME, United Nations Inter-agency Group for Child Mortality Estimation; WHO, World Health Organization.

a For all indicators, data or estimates are used by national governments and international agencies.

b CHAT technical advisory group recommends that this indicator be extended also to ages 5–9 years.

**TABLE 2.** Additional Indicators Recommended for High-Burden Countries

CHAT Core Indicator Name^a^	CHAT Recommended Definition	Country Data Source	Country Comparable Estimates?	SDG Indicator?	EWEC GS Indicator?
New HIV infections	Estimated number of new HIV infections per 1,000 uninfected population at risk of HIV infection	National and other surveys, facility data	UNAIDS (annual)	3.3.1	Yes
TB incidence	Number of new and recurrent (relapse) episodes of TB (all forms) occurring in a given year	Country notifications, prevalence studies	WHO Global TB Programme (annual)	3.3.2	Yes
Thalassemia prevalence	Birth prevalence of thalassemia	Country notifications (birth defect registries), prevalence studies	Estimates from WHO, IHME, and others	No	No
Use of insecticide treated bed-nets in children under-5 years	% children ages 0–59 months who slept under an insecticide-treated mosquito net the night prior to the survey	National and other surveys	No	No	Yes
Malaria diagnostics in children under-5 years	Proportion of children aged 0–59 months with fever in the last 2 weeks who had a finger or heel stick test	National and other surveys, facility data	No	No	No
Malaria treatment - first-line treatment for children under-5 years	% febrile children aged younger than 5 years receiving first-line antimalarial drug, among those receiving any antimalarial drug	National and other surveys, facility data	No	No	No

Abbreviations: CHAT, Child Health Accountability Tracking; EWEC GS, Every Woman Every Child Global Strategy; IHME, Institute for Health Metrics and Evaluation; SDG, Sustainable Development Goal; UNAIDS, Joint United Nations Programme on HIV/AIDS; WHO, World Health Organization.

a For all indicators, data or estimates are used by national governments and international agencies.

Tables 1 and 2 list the CHAT prioritized indicators with their data sources, reporting cycles, and uses at national and international levels to illustrate where data are coming from, how they are being used, and how national-level information systems are included in these measurement activities. Most low- and middle-income countries that use population-based surveys (i.e., Demographic and Health Surveys, Multiple Indicator Cluster Surveys) have data for multiple years for these indicators; alignment around this core set can improve health policy decision-making.

## INDICATORS CAPTURE DIFFERENT TYPES AND LEVELS OF INFORMATION AND SHOULD BE USED ACCORDINGLY

Improving the adoption and use of child health indicators for global monitoring requires a comprehensive understanding of the global data landscape and how country-level data come together to provide global and national values for an indicator at a given point in time. Table 3 presents the data collections used for reporting on the core indicators presented in Tables 1 and 2, along with the reporting cycles, sources, population coverage, strengths, and limitations of each. Each data collection captures different kinds of information—some representative of the national population and others specific to health facilities or specific disease/condition registries. Each has its strengths and weaknesses. For example, population-based surveys provide a snapshot of the results for indicators describing certain health outcomes, which are difficult to capture in routine health information systems. However, the long lag time of their reporting cycles means that current data are reflective only of past efforts. By contrast, routine health information systems provide country-level data that are continuously available from service and individual records systems for program monitoring, providing real-time data on the performance of health programs relative to population-based surveys. However, routine health information systems are only representative of the services provided at health facilities and the individuals who have access to these services. In addition, these systems suffer from data quality challenges and do not cover the whole health system.^13^ Notable gaps include private sector and community providers, making the data useful mainly for improving the quality of the services reporting into the system.^10^

**TABLE 3.** Types of Data Used for Indicators Monitoring Child Health and Well-Being

Data Collection	Reporting Cycle; Population Measured	Original Source	Strengths	Limitations	Link to Core Indicators and SDGs
CRVS	Annual; National population	National administrative records for births, deaths, and marriages	If registration is complete and the system functions efficiently, the data can be used to produce comparable country level estimates that are accurate and timely.	Costly to set up and maintain; in the absence of good coverage and completeness of CRVS data, may not cover the whole population or it could be incomplete.	Cause of death; inputs into under-5 mortality (3.2.1), mortality in children 5 to 9 years, birth registration (16.9)
Population-based surveys	3 to 5 years; National/subnational	National health surveys, DHS, MICS, censuses, malaria program surveys	Collect data that can't be obtained through other methods; provide population-based measures of coverage and health status; allows for equity analyses and can be disaggregated by a variety of different characteristics to describe the population of interest.	Conducted in-person in most LMICs, making them technically complex, expensive, and time consuming; reliance on respondents' self-report, which can add biases to the results; results reflect the survey reporting period with a 2-to-3-year time lag, so are not necessarily reflective of a country's current situation.	Cause of death; inputs into under-5 mortality (3.2.1), mortality in children 5 to 9 years; service coverage indicators: care seeking for acute respiratory infection and fever; diarrhea treatment; immunization (SDG 3.1); ECDI2030; use of insecticide-treated bed nets; maltreatment, harsh punishment by caregiver; vitamin A supplementation
Routine health information systems	Monthly; Facility or service specific	HMIS including DHIS2 and other platforms	Data are continuously available for program monitoring and provide a finer level of detail on the performance of specific health services within health facilities.	Data are only representative of the services provided through a health facility and only for those who seek care, leading to under-reported or biased coverage data. Many systems do not include services from the private sector or community providers. To create CHAT technical advisory group recommended indicators, these data would need to be used with another data source for a population-based denominator.	Administrative records systems (e.g., national health accounts), service records systems (e.g., immunizations administered, HMIS), and individual records systems (e.g., patient medical records), captured in an HMIS
Disease/condition registries, death audits	Monthly, annual; Facility, national	Disease/conditions specific registries, clearinghouses, death/disease audits	Captures diseases/conditions that are rarely reported; provides additional sources of data for rare conditions or uncommon events.	If facility based, may reflect only those seeking care in a facility; may not be representative of total population.	Registries (cancer, birth defects), surveillance systems; thalassemia prevalence, neural tube defect prevalence new HIV infections, TB incidence

Abbreviations: ARI, acute respiratory infection; CHAT, Child Health Accountability Tracking; CRVS; civil registration and vital statistics; DHS, Demographic and Health Survey; ECDI2030, Early Childhood Development Index 2030; HMIS, health management information system; LMICs, low- and middle-income countries; MICS, Multiple Indicator Cluster Survey; SDG, Sustainable Development Goal.

At an even higher level, country-comparable estimated values (e.g., estimates produced by the United Nations Inter-Agency Group on Child Mortality Estimation) derived from a variety of in-country data sources are a useful tool for global monitoring. They make it possible to compare outcomes and impact indicators across countries and over time. However, they are less useful locally or at a subnational level.^14^ Nonetheless, country-comparable estimates have become popular in the SDG era of target monitoring and are supported by global donors who use these to set their own funding agendas.^7^^,^^8^ Common concerns about estimated values include a lack of methodological transparency for some estimation efforts and the inclusion of input data of questionable quality or imputation methods based on assumptions that may not be valid.^14^ All of the data collection approaches in Table 3, taken together and used with full knowledge of their strengths and limitations, complement each other; they can be used to triangulate information on child health and well-being outcomes and monitor the impact of national and international efforts to improve primary health care and universal health coverage.

## FRAGMENTED M&E SYSTEMS REMAIN DESPITE SOME PROGRESS

Historically, vertical funding initiatives often resulted in parallel monitoring systems within a country, detracting from the ability of a country to assess the results of its overall health programming.^9^ M&E systems remain fragmented, with many focused on single disease programs (i.e., HIV, TB, immunization, or nutrition), legacies of past donor largesse. However, similarly to the integrated management of childhood illness movement that revolutionized management of the sick child in low- and middle-income countries, there is an opportunity to create health information management systems that cover all dimensions of child health and well-being in an integrated manner.

Those disease-specific programs that have been well funded enough to invest in data for their own M&E, use approaches that could be adapted for integrated M&E of child health outcomes through efforts to make better use of national data sources; provide training to national workforce; and promote transparency about data analysis methods, reporting cycles, limitations, and key uses in national and global platforms (Box 1).^9^^,^^15^^,^^16^ Furthermore, some initiatives that initially addressed single diseases have already taken steps toward integrating other areas, including reproductive, maternal, newborn, child, and adolescent health, leveraging United Nations agency and donor support to improve monitoring of health outcomes in an integrated manner (Box 2).^17^

BOX 1 HIV/AIDS: Building Country Data Collections in the Service of Global Monitoring of the HIV Epidemic Annually

Every year, the Joint United Nations Programme on HIV/AIDS (UNAIDS) provides revised global, regional, and country-specific modeled estimates using the best available epidemiological and programmatic data to track the HIV epidemic, including for children and adolescents. The data come mainly from country teams who use UNAIDS-supported software to develop estimates annually. The country teams, comprising national monitoring and evaluation specialists, program officers, epidemiologists, demographers, and others from ministries of health and national AIDS organizations, are trained by UNAIDS to use the Spectrum software (developed by Avenir Health) and its AIDS Impact Model to produce and collate their estimates.

The UNAIDS Reference Group on Estimates, Modelling and Projections provides technical guidance on the development of the HIV component of the AIDS Impact Model module. Other partners support the process by providing facilitators to country workshops, contributing data and ideas, and contributing to the costs of the workshops and capacity-building in countries.^16^ This united response to improving data on HIV/AIDS is a model for how the partnerships involved in child health could work together to strengthen data on childhood illness.

BOX 2 Moving From a Single Disease Focus to Integrated Monitoring for Improved Accountability for Reproductive, Maternal, Newborn, Child, and Adolescent Health in Countries and Globally

In 2009, the African Leaders Malaria Alliance, representing a coalition of African Union heads of state, came together to fight malaria. By 2011, African Leaders Malaria Alliance recognized the need for a scorecard to help monitor progress and strengthen accountability for malaria control and elimination. The scorecard accountability tool was updated quarterly with key indicators of country performance in controlling malaria for 46 malaria-endemic countries.^17^ The scorecards and quarterly reports were disseminated directly to heads of state and ministries of health and finance. This proved to be a popular tool for taking action against malaria. In 2012, the tools expanded their scope to support the development of reproductive, maternal, newborn, child, and adolescent health indicators and a neglected tropical disease tracer indicator. By 2020, 29 RMNCAH scorecards have been developed. These are updated annually and integrated into existing national management and decision-making processes. Quarterly scorecards can be downloaded here: https://alma2030.org/scorecard-tools/country-scorecards.

The primary aim of the scorecards is to strengthen national health information systems and improve accountability at the country level. A secondary result is improved reporting at the global level through better quality data aggregated to the national data for reporting on global targets. A sustainable method of producing annual or biannual child health and well-being scorecards for all countries off track to meet the Sustainable Development Goal child survival targets by 2030 would support countries in monitoring their progress.

## RECOMMENDATIONS FOR FUTURE GLOBAL MONITORING OF CHILD HEALTH AND WELL-BEING

Indicators, like those recommended by the CHAT (Tables 1 and 2), are often used in M&E systems based on a theory of change or logic framework. As a result of development frameworks that include targets and indicators for assessing progress—starting with the Millennium Development Goals and continuing with the SDGs—donors, governments, and international organizations have become more sophisticated and better at using indicators to report the results of their actions. M&E exercises are more integrated across diseases and conditions than in the past, making use of advances in technology and computing resources to capture information from multiple sources and for common risk factors.^9^

Many global initiatives could use existing data better to plan, implement, and monitor programs to improve child health and well-being. Given the opportunities to align investments and harmonize child health M&E approaches, we recommend that program managers and donor organizations make use of the dashboards and tools that are based on standard definitions and measurement approaches and created through consultative process (e.g., those generated by United Nations agencies like the World Health Organization and UNICEF and reviewed by technical advisory groups)^2^ to fully understand the wealth of data and associated indicators that have been validated and are currently in use for child health measurement.

Many global initiatives could use existing data better to plan, implement, and monitor programs to improve child health and well-being.

Over time, there will be a need for new and even revised indicators, particularly where measurement gaps exist and for areas of emerging concern. New indicators will be important for monitoring the Global Strategy for Women's, Children's and Adolescents' Health areas of “thrive” and “transform,” which measure child well-being, development, and how society is being transformed for the better by improvements in health and other sectors. Such indicators are multifaceted, often combining data from different sources. Effective coverage of interventions is an example of such an area that combines monitoring data from different sources to arrive at a fuller picture of how well children are able to access quality health services.^18^^,^^19^ Developing strong quality-of-care indicators for pediatric services while making better use of existing service coverage indicators will help us to transition from crude coverage measurement to effective coverage measurement to improve the quality and equity of service delivery.^18^

Although global measurement gaps are being tackled in multiple ways, national data collection and use needs further support. To improve country data collection, management, analysis, and use as the foundation of health system strengthening, we suggest the following 3 principles be applied to child health and well-being M&E frameworks going forward.

1. Avoid vertical child health M&E approaches because these can distort the prioritization of health issues at the country level and also make it difficult for national governments to allocate resources efficiently and effectively. Instead, promote integration of M&E systems at a country level so that there can be clear monitoring of overall progress on child health and well-being.
2. When developing national M&E systems to monitor and report on child health and well-being, use standard indicators that have been validated and recommended by normative agencies and are appropriate for the level of monitoring required.
3. Focus on building country capacity to improve child health data collection and analysis, thereby reducing reliance on estimation exercises to produce country-comparable estimates from multiple data sources. The use of estimates detracts funding and attention away from country efforts to improve M&E and data systems.

For monitoring at the outcome and impact levels, we have a validated collection of robust indicators that can be used for a variety of monitoring activities at the global and national level.^4^^,^^13^^,^^20^ In many cases, there is no need to develop more indicators in well-covered areas. Appropriate use of these current indicators—as signs of the need to gather more comprehensive information about a problem area and implement solutions—will go a long way toward improving child health and well-being. Quality, affordable, and equitably distributed health care will, in turn, help children develop into healthy adolescents and adults, well able to provide for the next generation.

## Notes

**Peer Reviewed**

**First published online:** December 6, 2023.
